# Oxidative Stability and Nanoencapsulation of Marine Omega-3 LC-PUFAs for Sustainable Aquaculture and Human Health

**DOI:** 10.3390/antiox15091184

**Published:** 2026-09-17

**Authors:** Ana Luísa Rebelo, Luís F. Baião, Marta Monteiro, Sofia A. Costa Lima

**Affiliations:** 1 Instituto de Ciências Biomédicas Abel Salazar, Universidade do Porto, Rua de Jorge Viterbo Ferreira 228, 4050-313 Porto, Portugal; 2 LAQV, REQUIMTE, Praça Coronel Pacheco nº15, 6º Andar, 4050-453 Porto, Portugal; 3 Sense Test Lda., Rua Zeferino Costa 341, 4400-345 Vila Nova de Gaia, Portugal; 4 CIIMAR/CIMAR LA, Centro Interdisciplinar de Investigação Marinha e Ambiental, Universidade do Porto, Av. General Norton de Matos S/N, 4450-208 Matosinhos, Portugal; 5 GreenUPorto—Sustainable Agrifood Production Research Centre/Inov4Agro, DGAOT, Faculdade de Ciências, Universidade do Porto, Campus de Vairão, Rua da Agrária 747, 4485-646 Vairao, Portugal

**Keywords:** lipid oxidation, oxidative stability, antioxidant protection, nanoencapsulation, omega-3 fatty acids, eicosapentaenoic acid, docosahexaenoic acid, long-chain polyunsaturated fatty acids, fish oil replacement, one health, sustainable aquafeeds

## Abstract

Eicosapentaenoic acid (EPA) and docosahexaenoic acid (DHA) are omega-3 long-chain polyunsaturated fatty acids (LC-PUFAs) that make farmed fish valuable to human health, given their bioactivity and highly unsaturated structure that also renders them exceptionally prone to oxidation during feed manufacture and storage, within the fish, and along the supply chain to the consumer. Preserving the oxidative stability, and hence the biological value, of these fatty acids is therefore a central challenge for sustainable aquaculture, made more acute by the replacement of omega-3-rich, oxidatively buffered fish oil with terrestrial oils that lower LC-PUFA content and shift the pro-/antioxidant balance of aquafeeds. Adopting an integrative feed-to-fish-to-consumer perspective, this review examines the strategies proposed to retain and protect EPA and DHA in farmed fish, distinguishing the physiological requirements of fish from the nutritional targets relevant to humans. We first appraise, comparatively, fish oil replacement, finishing diets and nutritional programming, showing that recovery of EPA and DHA is species-, tissue- and context-dependent and seldom complete, with EPA, a key eicosanoid precursor, preferentially catabolised. Through a focused, reproducible literature search, we then evaluate antioxidant-oriented nanoencapsulation as a means of shielding marine LC-PUFAs from oxidation and improving their stability and bioavailability. Although nanoencapsulation consistently enhances oxidative stability and, in the few in vivo studies available, fish performance, the evidence is constrained by heterogeneous and frequently incomplete physicochemical characterisation, scarce storage stability and in vivo data, and largely unaddressed safety, environmental-fate, regulatory, scalability and consumer-acceptance concerns. We conclude that protecting omega-3 oxidative stability will require combining well-characterised delivery systems with species-appropriate nutrition, and we set out the standardised characterisation, required in vivo validation, and integrated safety and life-cycle assessment needed to realise antioxidant-protective, nanotechnology-enabled aquafeeds.

## 1. Introduction

Eicosapentaenoic acid (EPA) and docosahexaenoic acid (DHA) are the omega-3 long-chain polyunsaturated fatty acids (LC-PUFAs) primarily responsible for the health value of farmed fish, yet the very feature that underlies their bioactivity, a highly unsaturated structure with multiple methylene-interrupted double bonds, also makes them exceptionally susceptible to oxidation. Their oxidative degradation begins in the feed and continues within the fish and along the supply chain to the consumer, eroding nutritional quality, generating potentially harmful oxidation products, and consuming the antioxidant reserves that protect cell membranes. Preserving the oxidative stability of EPA and DHA is therefore inseparable from preserving their nutritional benefit, and it is the unifying lens adopted throughout this review. The challenge has intensified as aquaculture has expanded and as omega-3-rich, oxidatively buffered fish oil has been progressively replaced by terrestrial oils that both lower LC-PUFA content and shift the pro-/antioxidant balance of aquafeeds.

Aquaculture yields fish, shellfish and algae for direct human consumption, as well as raw materials for the food industry, livestock nutrition, energy production, personal-care products, functional foods and biomedical applications [1]. The Food and Agriculture Organization of the United Nations (FAO) reports that, in 2022, aquaculture exceeded capture fisheries in aquatic animal production for the first time, reaching 94.4 million tonnes and contributing 51 percent of global output; nearly 89 percent of total fisheries and aquaculture production was destined for human consumption, while the remainder supported non-food sectors, primarily the manufacture of fishmeal and fish oil [2].

This expansion has been accompanied by more frequent aquatic disease outbreaks, which hinder sustainable growth and cause significant financial losses [3]. Antibiotics are often the first treatment option [4], yet their long-term use promotes bacterial resistance, harms fish health and the surrounding environment, and leaves residues in aquatic products. Vaccination reduces disease susceptibility but has limited effectiveness [5]. Against this background, the World Health Organization and the FAO advocate the prophylactic use of natural immunomodulators to enhance fish immunity and prevent disease, thereby safeguarding environmental, animal and human health [6].

Fatty acids (FAs) can act as such immunomodulatory agents, promoting immune competence, elevating antioxidant capacity, attenuating inflammation and increasing resistance to infection in aquatic animals [7]. Fish have a limited capacity to synthesise omega-3 LC-PUFAs and must obtain them from the diet, where they maintain cellular membrane integrity, regulate immunity and inflammation, and modulate metabolism [8]. Farmed fish were traditionally fed diets rich in fishmeal and fish oil; although this ensured an adequate supply of omega-3 LC-PUFAs, a strong dependence on finite marine stocks from capture fisheries is no longer sustainable [9]. Consequently, fishmeal and fish oil are now used selectively as strategic ingredients to maintain palatability, support growth and ensure minimum quality standards [10]. However, the growing use of plant- and animal-based substitutes raises concerns about fish health and welfare, because a weakened immune system increases vulnerability to disease and its economic consequences [11]. Terrestrial ingredients, chiefly plant oils from oilseeds, are rich in omega-6 and poor in omega-3 PUFAs, and therefore alter the FA composition of farmed fish [12]. A deterioration in feed, and hence fillet quality, can both undermine fish health and reduce the dietary intake of LC-PUFAs in humans, diminishing the overall benefits of aquaculture products [13]. The sector must therefore seek alternatives, whether through precise nutritional formulation or targeted supplements that boost LC-PUFA biosynthesis and/or retention via processes such as gene expression or enzyme activity [9].

Several recent reviews have addressed omega-3 nutrition, fish oil replacement and, separately, nanotechnology in aquaculture. The present review is distinguished by three considered choices. First, it is organised around the oxidative stability of EPA and DHA as a unifying lens within a feed-to-fish-to-consumer (One Health) framework, so that each strategy is judged not only by its effect on fish growth but by whether it protects and preserves the omega-3 LC-PUFAs ultimately delivered to human consumers. Second, rather than cataloguing findings, it foregrounds the inconsistencies and unresolved controversies in the field: the asymmetric handling of EPA and DHA, the species- and context-dependence of recovery, and the gap between promising in vitro nanocarrier performance and the near-absence of in vivo, safety and regulatory evidence. Third, antioxidant-oriented nanoencapsulation for the protection and delivery of marine LC-PUFAs, identified through a focused, reproducible search (Section 2), is treated as the core contribution and appraised critically rather than descriptively.

Accordingly, the objectives of this review are to: (i) define the physiological roles and requirements of LC-PUFAs in fish and distinguish these from the nutritional targets relevant to human health (Section 3); (ii) critically and comparatively evaluate current fish oil replacement strategies: alternative lipid sources, finishing diets and nutritional programming, together with their limitations (Section 4); and (iii) assess antioxidant-oriented nanoencapsulation and delivery as a means of protecting marine LC-PUFAs from oxidation and improving their stability and bioavailability, with particular attention to characterisation quality, in vivo validation, safety, environmental fate, regulation, scalability and consumer acceptance (Section 5). Section 6 integrates these threads into a prioritised research agenda.

## 2. Review Approach and Literature Search

This review followed a structured literature-search strategy designed to identify recent primary research on the delivery of long-chain omega-3 fatty acids (EPA and DHA) using nanoparticles and nanoencapsulated compounds in aquaculture and fish. The search was conducted in the PubMed and Scopus bibliographic databases and was restricted to peer-reviewed articles published between 2020 and 2026, so that the review reflects the most current evidence in this rapidly evolving field. PubMed provides authoritative, controlled-vocabulary coverage of the biomedical and nutrition literature, while Scopus adds broad multidisciplinary coverage, including the food-science, materials and engineering journals in which much nanoencapsulation work is published; together they give balanced coverage appropriate for an overview of current evidence.

The search was structured around three keyword combinations, joined by the Boolean operator “AND”: “aquaculture AND nanoparticles AND fatty acids”, “aquaculture AND nanoparticles AND immunity”, and “nanoencapsulation AND fatty acids”. Broad terms (“nanoparticles”, “nanoencapsulation” and “fatty acids”) were chosen deliberately to capture every study in which omega-3 fatty acids were nanoencapsulated within a particulate carrier, irrespective of carrier type. Narrower system-specific terms (e.g., “liposome”, “nanoemulsion”, “NLC”) or cargo-specific terms (e.g., “EPA”, “DHA”, “fish oil”) were avoided because they tend either to retrieve formulation-focused papers in which lipids are structural components of the particle rather than the delivered cargo, or to exclude relevant records that do not name the specific fatty acid.

Applying identical criteria to both databases, the three PubMed searches returned 20, 195 and 87 records, respectively, of which eight met the inclusion criteria; repeating the searches in Scopus under the same criteria returned nine eligible records, four of which had not already been retrieved by PubMed, so that, after the removal of duplicates, 12 direct LC-PUFA nanoencapsulation studies were retained. These 12 studies constitute the core evidence base for the nanoencapsulation analysis and are all listed in Table S1; the broader set of nanoparticle-in-aquaculture studies retrieved (addressing, for example, immune or growth outcomes) is drawn on for context in Section 5.1, and all included studies are listed in Supplementary Table S1, which distinguishes direct LC-PUFA nanoencapsulation studies from nanoparticle studies addressing other aquaculture applications. Studies were retained when they were original experimental works, in vivo or in vitro, that applied nanoparticles or nanoencapsulated compounds to the delivery of LC-PUFAs and reported antioxidant, oxidative-stability, growth-performance, fatty-acid or related physiological outcomes; aquaculture applications were prioritised. Records were excluded when they fell outside this scope, duplicated a study already captured by another search string, or had been retracted. For the purpose of Table S1 and this review, a system is considered “nanoscale” when it has at least one external dimension at the sub-micron scale (≤~1000 nm), and we distinguish nanoparticles (solid polymeric or inorganic particles), nanoemulsions (kinetically stable liquid-in-liquid dispersions, typically <200 nm), nanostructured lipid carriers (NLC; a solid–liquid lipid matrix) and micron-scale (>1 µm) systems.

## 3. The Role of Long-Chain Polyunsaturated Fatty Acids in Aquaculture

### 3.1. Chemical Structure and Oxidative Susceptibility

Fatty acids are organic molecules characterised by a long aliphatic hydrocarbon chain terminated by a carboxyl group at one end and a methyl group at the other [14]. In the body, they are typically esterified to glycerol as mono-, di- or triglycerides [15]. Long-chain fatty acids, defined as those with more than 12 carbon atoms, comprise saturated fatty acids (no double bonds), monounsaturated fatty acids (a single double bond) and PUFAs (two or more double bonds); PUFAs are further classified as omega-3 or omega-6 according to the position of the first double bond relative to the methyl end [16]. Beyond their structural role in the phospholipids of all cell membranes, FAs supply energy through β-oxidation and are stored in adipose tissue [17].

The multiple bis-allylic methylene positions of PUFAs make them particularly vulnerable to oxidation, and this vulnerability rises steeply with the degree of unsaturation, so that DHA (six double bonds) is even more oxidation-prone than EPA (five) [16,18]. Fish lipids therefore oxidise readily [19] when exposed to catalysts such as heat, light, metal ions and enzymes, which set off interconnected photo-, auto-, enzymatic and thermal oxidation pathways [20]. Autoxidation follows a classic free-radical chain mechanism: in initiation, a hydrogen atom is abstracted from a bis-allylic carbon to form a carbon-centred lipid radical; in propagation, this radical adds molecular oxygen to give a peroxyl radical that abstracts a further hydrogen, yielding a lipid hydroperoxide, the primary oxidation product, while regenerating the chain; and termination occurs when radicals combine or are quenched [21].

Lipid hydroperoxides are themselves odourless and tasteless but unstable, and their decomposition generates a spectrum of secondary oxidation products—short-chain aldehydes, ketones and other carbonyls such as malondialdehyde, 4-hydroxy-2-hexenal and propanal—that produce rancid off-flavours and odours and, as reactive electrophiles, can modify proteins, damage cell membranes and deplete endogenous antioxidant defences [20,21]. Because the chain is self-propagating, it continues until interrupted by antioxidants or other radical-scavenging mechanisms [21]; oxidative stability therefore reflects the balance between a feed’s pro-oxidant load and its antioxidant protection. This intrinsic instability is central to the present review: it is precisely the oxidative fragility of EPA and DHA, across feed processing, storage, digestion and tissue deposition, that both erodes the nutritional value ultimately delivered to consumers and motivates the antioxidant-protective encapsulation strategies discussed in Section 5.

### 3.2. Requirements and Functions of LC-PUFAs in Fish

EPA and DHA support a wide range of biological functions that vary considerably among tissues and species [22]. Freshwater species are generally able to biosynthesise LC-PUFAs from shorter-chain precursors, whereas marine species are largely unable to do so [13]. This metabolic limitation, together with the tendency of marine fish to store substantial omega-3 LC-PUFAs in their triacylglycerol reserves, underlines the critical importance of dietary LC-PUFAs for these species [23,24].

Essential fatty acid (EFA) requirements vary with species, developmental stage and environment, with early-life stages and broodstock being especially sensitive [25]. Even where some de novo synthesis occurs, the resulting LC-PUFA levels are often insufficient to meet the demands of fish under stress or the dietary needs of human consumers [13]. Requirement criteria should therefore extend beyond growth and survival to encompass fish health, ensuring EFA intakes adequate to sustain physiological homeostasis and proper immune responses [26]. The relevance of this broader criterion is illustrated by evidence that antiviral responses in salmonid cells depend strongly on cellular FA content, and that variations in EPA directly regulate numerous transcripts of the innate immune response during viral infection [27,28].

Context further shapes EFA requirements. Stressors such as hypoxia, which are increasingly prevalent under climate change, can alter omega-3 LC-PUFA demand [29], while water temperature modulates FA metabolism and energy storage in teleosts: colder water increases LC-PUFA synthesis and content to preserve membrane fluidity, whereas warmer water reduces LC-PUFA storage [30]. Because farmed fish are frequently exposed to crowding, confinement, hypoxia, handling and temperature fluctuation, their membrane composition, stress- and inflammation-related mediator production (for which EPA is a precursor) and overall EFA requirements are all elevated [12,13,23]. Dietary lipid quantity and balance are equally influential: a high omega-6/omega-3 ratio shifts membrane composition and eicosanoid signalling toward more intense and prolonged pro-inflammatory responses, weakening the moderating, anti-inflammatory influence of EPA-derived mediators and reducing stress resilience [31,32]. The increasing use of omega-6-rich vegetable oils in aquafeeds thus compounds the problem, driving membrane and eicosanoid profiles toward heightened stress reactivity.

For marine fish, salmonids and shrimp, omega-3 LC-PUFA requirements typically fall between 5 percent and 10 percent of total FAs (approximately 5–20 g/kg diet), depending on dietary lipid content, animal size and health status [23]. Dietary EFA deficiency produces clinical signs whose onset and severity are species-dependent: marine fish are typically affected within weeks, whereas salmonids may show delayed or minimal responses [24]. Taken together, these observations argue against a single universal requirement value and in favour of species- and context-specific EFA targets grounded in an understanding of the molecular and biochemical basis of PUFA metabolism. Critically, requirements defined for optimal fish health and growth need not coincide with those needed to preserve nutritional value for human consumption [33], developed in Section 3.4.

### 3.3. Factors Affecting Fatty Acid Retention

Among macronutrients, FAs are unusual in that they largely retain their original structure during digestion, so the body’s FA profile closely reflects dietary intake [14]. Diet is therefore decisive, but it is only one determinant among several. Metabolic pathways (selective incorporation, lipogenesis, β-oxidation) govern how individual FAs are processed and stored, with variation even between tissues; diet composition, feeding behaviour, fibre and anti-nutritional factors affect digestibility and deposition; and environmental factors such as temperature, salinity and season further modulate FA metabolism [34,35,36]. Fish, one of the most diverse vertebrate groups, consequently show substantial interspecific variation in FA composition [37].

**Figure 1:**
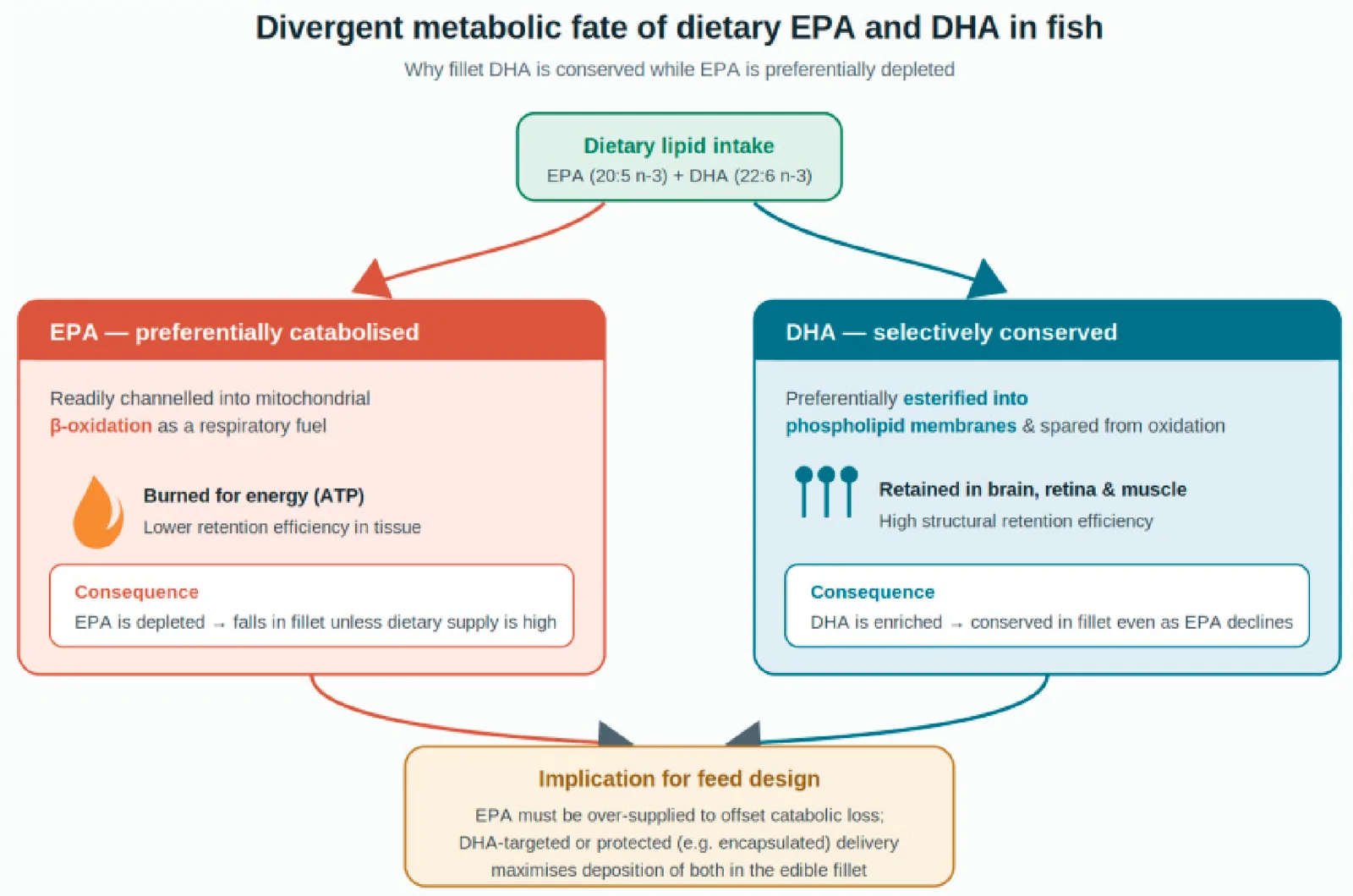
Divergent metabolic fate of dietary EPA and DHA in fish. EPA is preferentially routed to mitochondrial β-oxidation and depleted, whereas DHA is selectively esterified into phospholipid membranes and conserved, explaining why fillet DHA is retained as EPA declines and informing feed-formulation and delivery strategy. Conceptual synthesis based on Emery et al. [40].

This variability is not merely quantitative but mechanistic, and it carries a consistent and underappreciated asymmetry between EPA and DHA. Marine carnivores such as European sea bass retain LC-PUFAs efficiently, with reported muscle retention of 40–51 percent for EPA and 57–66 percent for DHA [38]. In Atlantic salmon, DHA retention is often in the range of roughly 30–60 percent, whereas EPA retention is more variable and frequently lower or inconsistent across diets [39]. The mechanism underlying this pattern is that EPA is preferentially catabolised as an oxidative substrate, while DHA is selectively conserved and retained in tissues (Figure 1) [40]. This EPA–DHA uncoupling is a recurring theme of the present review: it explains why fish oil replacement and finishing strategies so often restore DHA but not EPA (Section 4), and it implies that interventions must be evaluated separately for each FA rather than for total omega-3 content.

The same variability, compounded by the difficulty of defining exact EFA requirements, makes precise per-species FA reference values elusive. Nonetheless, indicative data exist: among wild marine fish, sardine and herring contain the highest EPA + DHA contents (25.6 and 16.8 g/kg wet weight, respectively), followed by Atlantic salmon (12.0 g/kg), whereas sea bass and cod contain only 2.3 and 2.1 g/kg [41]. These values differ markedly in farmed fish, where feeding regime and fat level vary widely, reinforcing that fillet FA composition is a manageable outcome of feed formulation rather than a fixed species trait. Reported EPA + DHA contents vary with wild-versus-farmed status, season and total lipid content, and other sources give lower figures for wild fish, for example, ~7.8 g/kg for wild Atlantic salmon and ~2.9 g/kg for wild sea bass [42,43], so the values above should be read as indicative rather than fixed.

Two distinct processes must be separated when interpreting fillet EPA and DHA levels. Metabolic loss (and, conversely, retention) refers to the in vivo fate of dietary LC-PUFAs, governed by dietary supply, selective β-oxidation (with EPA preferentially catabolised), elongation and desaturation, and tissue partitioning—and it is these processes, rather than chemical degradation, that principally explain the reduced fillet EPA and DHA observed after fish-oil replacement. Oxidative loss, by contrast, refers to the chemical degradation of EPA and DHA through lipid peroxidation in the feed and in tissues, which destroys the fatty acids and generates oxidation products. The two are distinct but interacting—oxidised dietary lipids can raise oxidative stress and secondarily perturb metabolism—and they call for different remedies: the nutritional strategies discussed in Section 4 act on metabolic retention, whereas the antioxidant and nanoencapsulation approaches discussed in Section 5 act on oxidative loss. This distinction is maintained throughout the review.

### 3.4. From Fish Physiological Requirements to Human Nutritional Targets

A central conceptual distinction, often unclear in the literature, is that the LC-PUFA levels required for fish health and growth are not the same as those required to make farmed fish a valuable dietary source for humans [25,33]. The two targets are set by different criteria, are expressed in different units, and can diverge substantially in practice; Table 1 makes this contrast explicit.

In human nutrition, omega-3 and omega-6 PUFAs are both vital, but their balance is critical. As in fish, a high intake of omega-6, or a high omega-6/omega-3 ratio, is associated with increased risk of cardiovascular, autoimmune, inflammatory and neoplastic disease, whereas higher omega-3 intake exerts anti-inflammatory effects [44,45]. Although humans can convert some α-linolenic acid to EPA and DHA, the conversion is limited and insufficient to meet requirements [46], so preformed dietary EPA and DHA, chiefly from oily fish, remain the primary source [47]. The European Food Safety Authority recommends 250 mg/day of EPA plus DHA for healthy adults, equivalent to one to two oily-fish meals per week [48]; notably, recommended intakes are not fully harmonised across authorities such as EFSA and the FDA [49], complicating the definition of a single fillet target.

Human dietary references are, moreover, expressed as combined EPA plus DHA rather than as separate values: neither EFSA nor the FDA defines an individual adult requirement for EPA and for DHA separately. Life-stage-specific guidance does exist—EFSA advises 100 mg DHA/day for infants and children up to two years, and an additional 100–200 mg DHA/day during pregnancy and lactation [50]. Because adult targets are combined, a strictly separate comparison of fillet EPA and DHA against human requirements is not currently possible, and the fillet targets discussed here are framed accordingly.

The consequence is a translational tension at the heart of sustainable aquafeed design. Salmonids, for example, can be reared on vegetable oils lacking omega-3 LC-PUFAs without impairing growth, yet this reduces the nutritional value of their flesh for consumers [25]. A feed that satisfies the fish may therefore fail the consumer. This leads to the question that motivates Section 4: if fish are a primary dietary source of LC-PUFAs, how can aquaculture ensure that farmed fish continue to deliver these health-promoting fatty acids?

**Table 1:** Contrasting reference frames for omega-3 LC-PUFAs: fish physiological requirements versus human nutritional targets. Values are indicative and drawn from the sources cited in the text; they illustrate the difference in criteria and units rather than defining thresholds.

Dimension	Fish Physiological Requirement	Human Nutritional Target
Primary criteria	Growth, survival, health, immune competence and stress resilience	Reduction of chronic-disease risk; adequacy of habitual intake
Typical expressed value	≈5–10% of total FAs (≈5–20 g/kg diet) for marine fish, salmonids and shrimp [23]	250 mg/day EPA + DHA for healthy adults ≈ 1–2 oily-fish meals/week [48]
Key modifiers	Species, life stage, temperature, hypoxia and other stressors, dietary n-6/n-3 ratio	Life stage, health status, background diet, non-harmonised guidance across authorities [49]
Key molecule(s)	EPA and DHA (EPA a key eicosanoid precursor; often catabolised)	EPA and DHA (preformed; endogenous ALA conversion insufficient)
Practical implication	Can be met on low-fish-oil diets in some species without growth loss	May not be met even when fish grow normally, if fillet EPA/DHA is low

## 4. Current Strategies for Fish Oil Replacement and Omega-3 Retention

Because EPA and DHA levels in the fillet matter for both fish and consumer health, replacement strategies must be judged against the dual, and not always aligned, criteria set out above. This section compares three broad approaches (Table 2): substitution with alternative lipid sources, finishing diets and nutritional programming. Across all three, a consistent pattern emerges: growth can usually be maintained without fish oil, but fillet EPA and DHA are difficult to preserve fully, and the shortfall is typically greater for EPA than for DHA.

### 4.1. Alternative Lipid Sources

Salmonids can tolerate complete replacement of fish oil with vegetable oils, and sea bass and sea bream show no growth impairment up to about 60 percent replacement; however, flesh omega-3 PUFAs fall by 50–65 percent [51]. In gilthead sea bream fed the highest replacement level, Menoyo et al. [52] reported that liver DHA fell roughly two-fold and EPA four-fold, with similar effects in muscle, an early, clear illustration of the EPA–DHA asymmetry introduced in Section 3.3.

Novel EPA and DHA sources are promising but constrained by high cost, conservation concerns, land-use competition and public resistance, particularly toward transgenic crops [53]. The primary study evidence is, moreover, genuinely contradictory, and these contradictions, rather than any single positive result, define the current state of the field. Full replacement of fish oil with soybean and linseed oils altered FA composition and induced lipid dysmetabolism in large yellow croaker [54], whereas DHA-rich canola oil performed well in Atlantic salmon [55]; the latter comparison is complicated, however, by incomplete fish oil substitution and by benchmarking against ordinary canola oil rather than fish oil, which limits direct comparison between the two studies. Microalgal oil (Veramaris^®^, Delft, The Netherlands) offers a more consistent counter-example, effectively substituting fish oil in gilthead sea bream [56] and rainbow trout [57] while maintaining growth, fillet EPA and DHA, health status and sensory quality, and reducing environmental impact and contaminant load. Against these successes, high inclusion levels (75–100 percent) of vegetable oils have repeatedly been associated with altered FA profiles, increased oxidative stress, impaired immunity and structural changes in the intestine and liver [58,59,60,61]. The balance of evidence therefore supports marine-microbial oils, but not generic vegetable oils, as robust one-for-one replacements, an important distinction that undifferentiated statements about “fish oil replacement” tend to obscure. These limitations motivate the complementary strategies below.

**Table 2:** Comparative overview of current strategies to preserve omega-3 LC-PUFAs in farmed fish. Entries summarise representative studies cited in the text; “outcome” and “limitation” are stated relative to fish continuously fed fish oil where applicable.

Strategy	Representative Studies (Species)	Intervention	Key Outcome	Main Limitation
Alternative lipid sources: vegetable oils	[51,52,54,58,59,60,61] (salmon, sea bream, sea bass, large yellow croaker)	Partial-to-full substitution of fish oil with plant oils	Growth often maintained; fillet omega-3 reduced by ~50–65%; EPA lost more than DHA	Oxidative stress, immune impairment and tissue changes at high inclusion; consumer nutritional value reduced
Alternative lipid sources: microbial/algal oil	[55,56,57] (rainbow trout, sea bream, Atlantic salmon)	Replacement with EPA/DHA-rich microalgal or DHA-canola oil	Growth, fillet EPA/DHA, health and sensory quality maintained; lower contaminant load	Cost, supply scale; some studies use incomplete substitution or non-fish-oil controls, complicating comparison
Finishing diets	[62,63,64,65,66] (rainbow trout, sea bass, sea bream, tiger puffer)	Fish-oil finishing diet after a vegetable-oil grow-out period	DHA largely restored; EPA often not fully recovered even after 90 days	Recovery depends on species/size/duration/tissue; reinstates dependence on marine oil
Nutritional programming	[67,68,69,70,71,72] (mainly gilthead sea bream, Atlantic salmon)	Early-life/broodstock vegetable-oil stimulus to improve later diet utilisation	Improved growth and lipid utilisation on later low-fish-oil diets in responsive species	Limited by poor C18 → LC-PUFA bioconversion in marine fish; heterogeneous protocols; not generalisable

### 4.2. Finishing Diets

Because growth can be sustained on vegetable-oil-rich diets, a fish-oil “finishing” diet is often applied after the grow-out period to restore fillet omega-3 LC-PUFAs and meet consumer expectations [62]. The underlying dilution model predicts that, after a sufficient feeding period, tissue FA composition converges toward that of the finishing diet as pre-existing reserves are diluted [63]. In practice, finishing diets restore much of the DHA content relative to fish continuously fed fish oil, but success depends on species, size, feeding duration, and FA and tissue type [64], and, once again, EPA is the harder FA to recover. In European sea bass, EPA and DHA were not fully restored after 20 weeks of a 100 percent anchovy-oil finishing diet, with fish reaching only about 70 percent of the levels of those fed fish oil continuously [65]. In gilthead sea bream, 60–80 percent substitution of anchovy oil reduced muscle DHA and, more markedly, EPA; reintroducing a 100 percent anchovy-oil diet recovered muscle DHA within 60 days but failed to recover EPA even after 90 days [66]. Finishing diets thus mitigate, but do not eliminate, the EPA deficit, and they reintroduce dependence on marine oil at the finishing stage.

The persistent shortfall in EPA also warrants a note of caution about strategy. The limited restoration of muscle EPA probably reflects inherent biological regulation, preferential retention, selective β-oxidation and tissue-specific distribution, rather than a simple dietary shortfall, so that muscle EPA may approach an adaptive set-point that further supplementation cannot easily raise [26,73]. From a human-nutrition standpoint, moreover, 250 mg/day of EPA plus DHA is considered sufficient for cardiovascular and neurological health, so pursuing excessively high fillet EPA is likely to be neither biologically efficient in the fish nor nutritionally necessary for the consumer. Maintaining current EPA and DHA levels, rather than seeking over-supplementation, may therefore be a more realistic and appropriate goal for finishing diets.

### 4.3. Nutritional Programming

Nutritional programming exploits windows of high developmental plasticity: brief early exposure to vegetable-based diets is used to improve later uptake and utilisation of nutrients when a comparable diet is reintroduced [67], producing fish that are better able to use plant meals and oils and, potentially, to compensate for low dietary omega-3 LC-PUFAs [68]. Industrially, the approach has attracted interest as a means of improving feed efficiency, growth and disease resistance [69]. Its most consistent, long-term benefits have been demonstrated in gilthead sea bream, where broodstock and early-life vegetable-oil stimuli repeatedly improved offspring growth and lipid utilisation on later low-fish-oil diets [70,71].

The broader relevance of this strategy is, however, questionable. Marine fish have a very limited capacity to bioconvert C18 precursors into omega-3 PUFAs and therefore require them preformed, a fundamental obstacle when fish oil is withdrawn [74]; success is thus likely confined to particular species. Two further limitations constrain interpretation. First, the literature spans highly heterogeneous experimental conditions: dietary approach, timing and duration of the challenge, which hinders generalisable conclusions [72]. Second, most studies manipulate a single nutrient class, yet dietary changes may shift other macro- or micronutrients, so overall nutritional balance must be considered [72]. Nutritional programming is therefore best regarded as a species-specific adjunct rather than a stand-alone solution, which, together with the residual EPA deficits of the other strategies, motivates the protective, delivery-oriented approach of Section 5.

## 5. Nanotechnology and Encapsulation for LC-PUFA Stability and Delivery

### 5.1. Rationale and Current Applications in Aquaculture

Nanotechnology concerns the design, development and use of materials at the nanometre scale, which may take the form of nanoparticles, nanoemulsions, nanofibres and others [75]. Their significance stems from size-dependent physicochemical behaviour arising from surface and quantum effects [76,77]: a large surface-area-to-volume ratio and tunable chemistry make nanoparticles useful across biotechnology, biomedicine, food and environmental technology [78], and their chemical stability and capacity for self-assembly allow them to be tailored to specific functions [79]. These properties make nanocarriers a plausible means of protecting FAs from oxidation while enhancing their stability and the nutritional quality of aquafeeds—directly addressing the oxidative fragility identified in Section 3.1.

In aquaculture, nano-solutions are already applied to fish health management and feed formulation [80], including antibacterial and antifungal nanostructured surfaces, nanosensors for pathogen detection, and targeted delivery of therapeutics through feed [81]. In feeds specifically, nanotechnology can improve the bioavailability, stability and controlled release of vitamins and minerals, thereby enhancing growth, feed efficiency, antioxidant status, immunity and nutrient retention [82]. Selenium nanoparticles improve growth and immune response [83], and nanoencapsulation of ascorbic acid preserves its stability and efficacy [84,85]. Against this active backdrop, the targeted nano-delivery of LC-PUFAs remains conspicuously underexplored.

It is also useful to situate nanoencapsulation among the conventional strategies already used to protect LC-PUFAs during feed manufacture and storage. These rely mainly on lipid-soluble antioxidants such as tocopherols [86,87], phenolic extracts and other radical scavengers [19,88], metal chelators (e.g., EDTA, citrates) that limit pro-oxidant transition metals [89], and control of oxygen, light, temperature and moisture during processing, often combined with optimised extrusion and coating. Such measures delay the initiation and propagation of lipid oxidation and are standard in commercial feeds. Nanoencapsulation is best viewed as complementary to, rather than a replacement for, these tools: polymeric or lipid-based nanocarriers add a physical barrier that isolates LC-PUFAs from oxygen, pro-oxidants and light, and can additionally improve retention under gastric conditions and enable targeted intestinal release, functions that bulk antioxidants do not provide.

### 5.2. Evidence for Nanoscale Protection and Delivery of LC-PUFAs

The focused search described in Section 2 identified a small and heterogeneous body of work (Table 1). Only one study addressed the nano-delivery of LC-PUFAs directly in fish aquaculture: Ibrahim et al. [90] reported that omega-3 nanoparticles enriched Nile tilapia flesh with omega-3 LC-PUFAs in a dose-dependent manner and improved growth, immunity and resistance to *Aeromonas hydrophila*. These in vivo results are encouraging, but the study provides no information on the nanoparticles’ physicochemical characteristics or storage stability, which critically limits interpretation: without storage-stability data, it cannot be established whether the system remains functional over time, a prerequisite for industrial use. The remaining studies successfully formulated nanosystems based on chitosan, proteins, modified agar/carrageenan and lipid carriers that protected PUFAs from oxidation but were conducted outside an aquaculture context [91,92,93,94,95,96,97,98,99,100,101].

Beyond these specific reports, nanotechnology is increasingly applied across aquaculture nutrition, health management, water-quality control and diagnostics [75,81,102,103], and micro- and nanodelivery systems have long been explored to improve the stability and bioavailability of marine bioactive lipids more generally [104]. Nanoparticles used in aquaculture can be grouped into inorganic, polymeric, lipid-based, chitosan-based and hybrid systems, each with distinct advantages and drawbacks, and are used to deliver nutrients, bioactive compounds, vaccines and therapeutics [85,105,106]. For heat- and shear-sensitive formulations (e.g., liposomes, solid lipid nanoparticles and chitosan-coated lipid nanoparticles), post-extrusion coating can better preserve particle structure, encapsulation efficiency and bioactive stability, whereas pre-extrusion incorporation may suit thermally robust or spray-dried systems; direct comparisons, however, remain scarce, and the choice must be validated per formulation [75,107].

### 5.3. Critical Appraisal: Characterisation and Reporting Gaps

The most important conclusion from this evidence is methodological. The reviewed formulations are so heterogeneous in size, zeta-potential and composition, and so frequently incomplete in their reporting, that direct comparison is not possible, and no system can currently be judged superior. Apparent differences in performance may reflect formulation variability or incomplete characterisation rather than genuine superiority, or indeed the intrinsic difficulty of encapsulating and delivering marine PUFAs. Three parameters persist as decisive yet under-reported. Encapsulation efficiency was highest for zein-protein nanoparticles [95], but that study reported neither zeta-potential nor storage stability and produced comparatively large particles. Indeed, although that study describes the production of nanocapsules, the kafirin wall material yielded particles of ~1.1 µm that fall outside the nanoscale (see the definition in Section 2), and this formulation is flagged accordingly in Table 3. Particle size governs not only absorption but colloidal stability, processing behaviour and storage performance: smaller particles enhance dispersion and bioavailability yet are more prone to aggregation and size change, which affects feed manufacture and long-term incorporation [108]. Zeta-potential predicts colloidal stability and aggregation tendency [109] and, because surface charge shapes interaction with immune-cell membranes and cellular uptake, also conditions any immunomodulatory function [110,111]; its frequent omission prevents evaluation of both stability and immunostimulatory potential. Storage stability, whether a system remains functional over time, was likewise unreported in several studies. The field’s priority, therefore, is not more formulations but complete, standardised characterisation: at minimum size, PDI, zeta-potential, encapsulation efficiency and storage stability reported together.

### 5.4. Translational Barriers: In Vivo Validation, Safety, Environment, Regulation, Scale and Acceptance

Even a well-characterised nanocarrier faces substantial barriers to real-world use, and these, rather than in vitro performance, will determine whether nanotechnology reaches commercial aquafeeds.

In vivo validation. Nanoparticle behaviour cannot be fully predicted from in vitro data, so fish trials are essential to assess real uptake, distribution, accumulation, effects on the immune system and the whole organism, and possible toxicity under realistic biological and environmental conditions [112]. The stability of the final pellets in different aquatic environments and of the nanosystems under actual feed-production conditions must also be tested. Current evidence rests largely on short-term laboratory exposures and non-standardised protocols, making it difficult to judge efficacy, stability, bioaccumulation, environmental-fate and food-safety implications under commercial feed processing, long-term feeding, species-specific husbandry, water-quality variation and the complete production cycle [106,113].

Safety and toxicity. Nanoparticle effects vary with size, surface charge, coating, dose and exposure route; fish may show oxidative stress, immune effects, tissue damage or reproductive effects depending on the formulation [114,115]. The absence of toxicity and biosafety data in the LC-PUFA delivery studies reviewed is a serious gap given that these carriers are intended for repeated dietary administration.

Environmental fate. Nanoparticles can aggregate, dissolve, transform, bioaccumulate and move through trophic levels, and their real behaviour in water remains insufficiently understood [114,115]. A life-cycle assessment (LCA) is warranted to establish whether nanoparticle-based aquafeeds are environmentally beneficial, quantifying impacts from synthesis through feed manufacture, use, excretion and end-of-life; conventional LCA should be complemented by nanomaterial-specific fate, exposure and ecotoxicity assessment to capture particle transformation and release [116].

Regulation, scalability and cost. There is still no dedicated regulatory framework for nanotechnology in food or aquaculture; existing provisions cover only general aspects and cannot guarantee safe use [80]. Manufacturing constraints such as process stability, quality control, difficult scale-up and high cost further limit application in aquafeed production [82].

These manufacturing constraints translate into a cost–benefit question that will ultimately govern adoption. The economic feasibility of nanoencapsulation depends on whether the value of the additional EPA and DHA retained exceeds the extra costs of carriers, encapsulation processing and quality control. Industrial fish oils and premixes are relatively inexpensive compared with functional polymers (e.g., PLGA, chitosan derivatives) and energy-intensive processing [117], so encapsulation is most likely to be justified in high-value applications, larval feeds, functional or nutraceutical products, and products with extended shelf-life or stringent oxidative specifications, where modest gains in retention carry measurable performance or market value [118]; for commodity grow-out feeds, it may not currently be cost-effective.

Consumer acceptance. Because consumers ultimately steer the industry, their willingness to eat fish reared on nanotechnology-enhanced feeds is decisive [80]. Studies specifically addressing this are scarce, but nanotechnology is more readily accepted in novel packaging with clear benefits than when applied directly to food [119]. These concerns should be treated as a research agenda rather than a barrier: nanotechnology remains a highly promising route to protecting and delivering bioactive compounds such as LC-PUFAs, and the gaps identified here define the work needed to realise it responsibly.

## 6. Conclusions and Future Perspectives

Viewed through the feed-to-fish-to-consumer lens adopted here, the evidence supports one overarching conclusion: no single strategy can currently restore EPA and DHA across aquaculture species, and the recurrent, mechanistically grounded reason is the preferential catabolism of EPA relative to DHA, which undermines both replacement and finishing approaches. Alternative lipid sources maintain growth but, except for marine-microbial oils, reduce fillet omega-3; finishing diets recover DHA more reliably than EPA and reinstate marine-oil dependence; and nutritional programming benefits only responsive, mostly bioconversion-capable species. Nanotechnology offers a complementary, protection-and-delivery approach whose in vitro promise is real but whose translational evidence is not yet in place.

Realising that promise requires a specific and prioritised research agenda. We propose the following: (i) map species- and context-specific EFA requirements: distinguishing EPA from DHA and physiological from consumer-nutritional targets before optimising any delivery system; (ii) adopt standardised, complete reporting of nanocarrier characterisation, minimally comprising particle size, PDI, zeta-potential, encapsulation efficiency and storage stability, to enable cross-study comparison; (iii) make in vivo validation in the target species mandatory, with pre-defined endpoints spanning retention, growth, immune function, tissue accumulation and toxicity, under realistic feed-processing and husbandry conditions; (iv) integrate nanomaterial-specific safety, bioaccumulation, environmental-fate and LCA into formulation development rather than treating them as afterthoughts; (v) develop the regulatory frameworks and scalable, cost-controlled manufacturing routes needed for industrial adoption; and (vi) conduct consumer-acceptance research specific to nanotechnology-enhanced feeds. Because the strategies reviewed are individually incomplete, the most plausible route to nutritionally adequate, sustainable farmed fish is a combination of complementary approaches: an oxidatively protected, well-characterised LC-PUFA delivery system deployed alongside species-appropriate nutritional strategies and tailored to each production context.

## Figures and Tables

**Table 3:** Overview of published encapsulated-PUFA formulations, their key characteristics, main outcomes and principal reporting gaps.

Reference	Cargo	Nanosystem/Method	Size/PDI/ZP	EE	Stability	Main Outcome & Key Gap
[91]	Refined cobia liver oil	Chitosan–sodium tripolyphosphate; emulsification + ionic gelation	~174–456 nm/0.22–0.27/+15–35 mV	~25–50%	Improved	Chitosan encapsulation improved oxidative stability; not tested in fish
[90]	Omega-3 PUFAs	Sodium alginate–acrylic acid; gamma irradiation	No data	No data	No data	In vivo benefits to tilapia growth/immunity; no particle characterisation or stability
[93]	PUFA concentrates from tuna oil	Chitosan; emulsification	~220 nm/<0.30/not specified	No data	Stable 30 d at 7 °C	Oxidative stability shown; EE and ZP not reported
[95]	Fish oil	Kafirin or zein proteins; monoaxial electrospraying	Kafirin ~1.1 µm */0.21/no data	Kafirin ~40%; zein ~93%	No data	Highest EE (zein) but large size; no ZP or storage-stability data
[100]	DHA	Low-melting-point modified agar (LAG) or κ-carrageenan (KC); anti-solvent	LAG ~313 nm/0.31/−29.23 mV	LAG ~67%; KC ~61%	Stable 21 d	Good encapsulation and stability; not validated in feed/fish
[101]	Mullet oil	Cocoa butter/mullet UFAs/Tween 80 (nanoemulsions & NLC)	NE ~170–406 nm/0.20–0.3	No data	No data	Suitable size and dispersion; EE and storage stability not reported
[94]	Shrimp oil	Lecithin; ultrasonication (A)/microfluidization (B)	40–284 nm (A); 214–928 nm (B)	94% (A); 75% (B)	Stable 8 w at 30 °C	Oxidative stability shown; ZP not reported
[98]	Tuna fish oil	Modified starches with curcumin and resveratrol; ultrasonication	Not specified/0.13–0.25/−33 to −19 mV	81–96%	1 month	Particle size not specified
[92]	Omega-3 FAs (garfish skin oil)	Sodium alginate–acacia gum complex	962 nm/0.506/−40.3 mV	90%	Stable 90 d	Large size and high PDI
[97]	Omega-3 FAs	Whey protein isolate + polyvinyl alcohol; electrospinning	263 nm	91–98%	No data	Enhanced stability; ZP/PDI not reported
[99]	DHA	Chitosan; ionic gelation	77 nm/0.36/+22 mV	83%	No data	Enhanced stability; storage stability not reported
[96]	Seabass oil	Chitosan–tripolyphosphate; emulsification + ionic cross-linking	115–318 nm/0.15–0.24/+4 to +41 mV	33–63%	8 weeks	No in vitro release, bioavailability or cytotoxicity data

“No data” indicates the parameter was not reported in the source. PDI, polydispersity index (a Dynamic Light Scattering measure of size-distribution breadth); EE, encapsulation efficiency; ZP, zeta-potential. * The kafirin formulation lies outside the nanoscale. Methods for lecithin nanosystems: (A) ultrasonication, (B) microfluidization.

## Data Availability

No new data were created or analysed in this study. Data sharing is not applicable to this article.

## Supplementary Materials

The following supporting information can be downloaded at: https://www.mdpi.com/article/10.3390/antiox15091184/s1, Table S1: Studies retrieved by the literature search (Section 2), classified as direct LC-PUFA nanoencapsulation studies versus nanoparticle studies addressing other aquaculture applications.
