# Supplementary material for: Oxidative Stability and Nanoencapsulation of Marine Omega-3 LC-PUFAs for Sustainable Aquaculture and Human Health
Source: Antioxidants (Basel). 2026 Sep 17;15(9):1184. doi: 10.3390/antiox15091184 (PMC13603150; doi:10.3390/antiox15091184)
Supplement: Supplementary file 1 [file antioxidants-15-01184-s001.zip › antioxidants-4560486-supplementary.pdf]

antioxidants

MDPI

Review

Oxidative Stability and Nanoencapsulation of Marine Omega-3 LC-PUFAs for Sustainable Aquaculture and Human Health

Table S1. Studies retrieved by the literature search (Section 2), classified as direct LC-PUFA nanoencapsulation studies versus nanoparticle studies addressing other aquaculture applications.

#	Study	Cargo	Nanosystem / method	Retrieved by (search string / database)
1	**Gulzar & Benjakul (2020)**	Shrimp oil	Lecithin nanoliposomes; ultrasonication / microfluidization	*nanoencapsulation AND fatty acids (PubMed, Scopus)*
2	**Shehzad et al. (2021)**	Tuna fish oil	Modified-starch nanoemulsions + curcumin/resveratrol; ultrasonication	*nanoencapsulation AND fatty acids (Scopus)*
3	**Chang et al. (2021)**	Refined cobia liver oil	Chitosan–sodium tripolyphosphate; emulsification + ionic gelation	*nanoencapsulation AND fatty acids (PubMed,Scopus)*
4	**Ibrahim et al. (2022)**	Omega-3 PUFAs	Sodium alginate–acrylic acid; gamma irradiation	*aquaculture AND nanoparticles AND fatty acids / immunity (PubMed,Scopus)*
5	**Engelmann et al. (2024)**	PUFA concentrates (tuna oil)	Chitosan; emulsification	*nanoencapsulation AND fatty acids (PubMed,Scopus)*
6	**Rahmani-Manglano et al. (2024)**	Fish oil	Kafirin or zein proteins; monoaxial electrospraying	*nanoencapsulation AND fatty acids (PubMed)*
7	**Z. Du et al. (2025)**	DHA	Low-melting-point agar (LAG) or κ-carrageenan; anti-solvent	*nanoencapsulation AND fatty acids (PubMed)*
8	**Devi et al. (2025)**	Omega-3 FAs (garfish skin oil)	Sodium alginate–acacia gum complex	*nanoencapsulation AND fatty acids (Scopus)*
9	**Saghaee et al. (2025)**	Omega-3 FAs	Whey protein isolate + PVA; electrospinning	*nanoencapsulation AND fatty acids (PubMed)*
10	**Quadros et al. (2026)**	Mullet oil	Cocoa butter / mullet UFAs / Tween 80; nanoemulsions & NLC	*nanoencapsulation AND fatty acids (PubMed,Scopus)*
11	**Vijayakumar & Sankaran (2026)**	DHA	Chitosan; ionic gelation	*nanoencapsulation AND fatty acids (Scopus)*
12	**Sae-leaw & Benjakul (2026)**	Seabass oil	Chitosan–tripolyphosphate; emulsification + ionic cross-linking	*nanoencapsulation AND fatty acids (Scopus)*

* The three keyword combinations ("aquaculture AND nanoparticles AND fatty acids", "aquaculture AND nanoparticles AND immunity", and "nanoencapsulation AND fatty acids") were run in PubMed and Scopus for 2020–2026 (see Section 2). The immunity-axis searches returned large numbers of records but, after screening against the inclusion criteria, yielded no additional eligible LC-PUFA-delivery studies beyond those in the table
